# Basal cell carcinoma in dark skin: clinical and dermoscopy features^؆^

**DOI:** 10.1016/j.abd.2026.501433

**Published:** 2026-08-07

**Authors:** Priscila Jordana Costa Valadares, Maria Luisa Pires de Freitas, Diego Guimarães Florêncio Pujoni, Flávia Vasques Bittencourt

**Affiliations:** a Dermatology Service, Hospital das Clínicas, Universidade Federal de Minas Gerais, Belo Horizonte, MG, Brazil; b Institute of Biological Sciences, Universidade Federal de Minas Gerais, Belo Horizonte, MG, Brazil; c Department of Dermatology, Faculty of Medicine, Universidade Federal de Minas Gerais, Belo Horizonte, MG, Brazil

*Dear Editor,*

Basal Cell Carcinoma (BCC) is the most common skin cancer worldwide; however, its clinical and dermoscopic features in darker phototypes remain underexplored. Research predominantly based on lighter skin tones may hinder the recognition of BCC in individuals with skin of color, in whom pigmentation often masks classical diagnostic features such as translucency and telangiectasias.^1^ In these patients, dermoscopy becomes an essential diagnostic tool, as studies have shown that BCC in skin of color frequently presents as pigmented lesions that may mimic other benign or malignant conditions.^2^, ^3^ Additionally, the underrepresentation of darker phototypes in BCC-related clinical studies ‒ fewer than half of which report participants’ skin color or phototype ‒ limits the applicability of existing diagnostic frameworks.^4^

This study aimed to characterize the epidemiological, clinical, dermoscopic and histopathological aspects of BCCs in Brazilian patients with Fitzpatrick skin phototypes IV and V.

A retrospective analysis was performed on 76 histopathologically confirmed BCCs from 63 patients. Images were acquired using Canon PowerShot, Nikon 1, DermLite DL-3/DL4, or Fotofinder dermoscopes. Three dermatologists evaluated the images together and reached a consensus on each lesion’s characteristics.

Among the 63 patients, 30 were female and 33 male, with a mean age of 70-years (range 49–84). The clinical characteristics of the tumors are summarized in Table 1.

**Table 1:** Clinical aspects of basal cell carcinomas.

Location		Size		Macroscopic pigmentation	
Total	76 (100%)	Total	76 (100%)	Total (of the surface area)	76 (100%)
Head/neck	60 (79%)	>10 mm	33 (43%)	Very pigmented (>70%)	39 (51%)
Trunk	11 (14%)	10‒5 mm	23 (30%)	Pigmented (30%‒70%)	11 (14%)
Upper limbs	3 (4%)	<5 mm	20 (26%)	Lightly pigmented (<30%)	22 (29%)
Lower limbs	2 (3%)			Non-pigmented	4 (5%)

Histopathologically, 37 (49%) of the BCCs were classified as nodular, 19 (25%) as superficial, 2 (3%) as infiltrative, and 15 (20%) exhibited mixed histology. The subtype could not be determined in 3 (4%) cases.

Regarding the proportion of the tumor area that showed pigmentation under dermoscopy, 34 (45%) lesions were highly pigmented (>70% of the surface area), 17 (22%) were moderately pigmented (30%–70% of the surface area), 24 (31%) were lightly pigmented (<30% of the surface area), and only one (1%) showed no pigmentation. Table 2 summarizes the frequency of the other dermoscopic features across all tumors and compares their distribution between nodular and superficial subtypes.

**Table 2:** Dermoscopic features of basal cell carcinomas.

Type	Basal cell carcinoma	Nodular basal cell carcinoma	Superficial basal cell carcinoma
Number of lesions	76 (100%)	37 (100%)	19 (100%)
Pigmentation	75 (99%)	36 (97%)	19 (100%)
Leaf-like areas	44 (58%)	18 (49%)	17 (89%)
Large blue-grey ovoid nests	23 (30%)	14 (38%)	0 (0%)
Multiple blue-grey globules	31 (41%)	11 (30%)	8 (42%)
Multiple in-focus blue-grey dots	16 (21%)	6 (16%)	3 (16%)
Spoke wheel areas	9 (12%)	2 (5%)	5 (26%)
Concentric structures	4 (5%)	2 (5%)	2 (11%)
White/red structureless areas	19 (25%)	3 (8%)	12 (63%)
Ulceration	34 (45%)	14 (38%)	6 (32%)
Multiple small erosions	12 (16%)	2 (5%)	6 (32%)
Arborizing vessels	24 (32%)	16 (43%)	0 (0%)
Short fine telangiectasias	5 (7%)	2 (5%)	3 (16%)
Other vessels	9 (12%)	4 (11%)	1 (5%)

Compared with previous reports, the proportion of pigmented BCCs in this series was markedly higher, consistent with findings from non-Caucasian populations.^5^, ^6^ Highly pigmented lesions (>70% of the surface pigmented) accounted for 45% of cases, whereas among pigmented BCCs in lighter skin tones, only 7%–13% are highly pigmented.^7^ This contrast reinforces pigmentation as a key diagnostic feature of BCC in darker phototypes.

Leaf-like areas were the most frequent dermoscopic structure, appearing in 58% of lesions ‒ higher than frequencies described in most prior studies, with a comparable finding only in an Indian population of similar phototype.^8^, ^9^ This finding suggests that leaf-like areas may serve as an important diagnostic clue for BCC in darker skin. The predominance of pigmentation may obscure classical dermoscopic features such as arborizing vessels and short fine telangiectasias, which were less frequently detected in this study.

The data on pigmented structures were also compared with a systematic review published in 2021 by Reiter et al., which quantified the prevalence of dermoscopic structures present in BCCs.^10^ Only the leaf-like areas were significantly more prevalent in our study compared to the systematic review (p < 0.05; Fig. 1). This result may seem unexpected, given the much higher degree of pigmentation observed in our cohort. However, this apparent discrepancy can be explained by the presence of diffuse pigmentation on dermoscopy in 19 out of 76 tumors (25%), making it difficult ‒ or even impossible ‒ to identify specific pigmented structures (Fig. 2). Such a dense pigmentation pattern poses a diagnostic challenge, as these lesions may mimic blue nevus, melanoma, or vascular tumors. The dense pigment, often lacking recognizable dermoscopic architecture, also hinders visualization of non-pigmented structures.

**Fig. 1:**
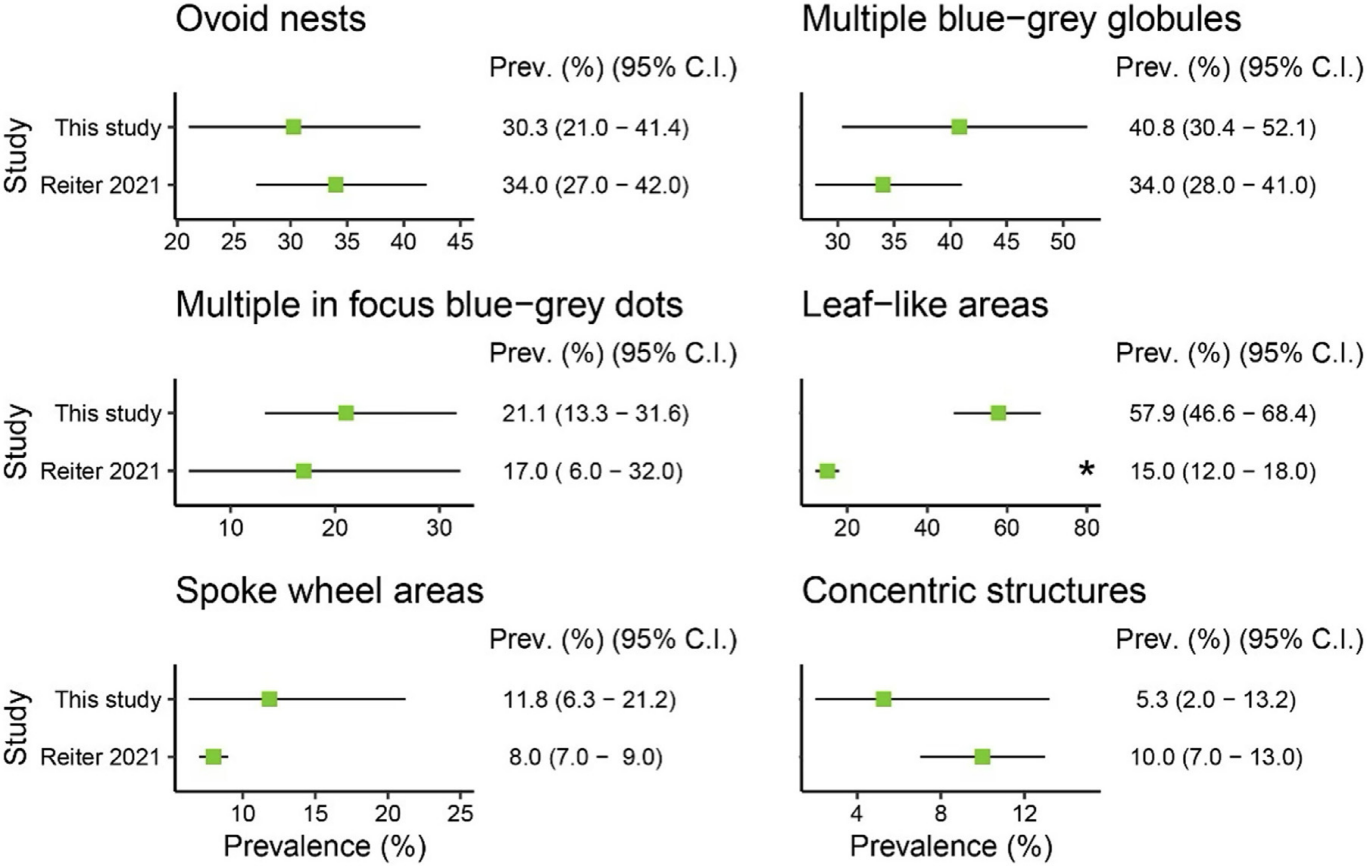
Prevalence of pigmented dermoscopic structures in basal cell carcinoma: comparison with a systematic review.^10^

**Fig. 2:**
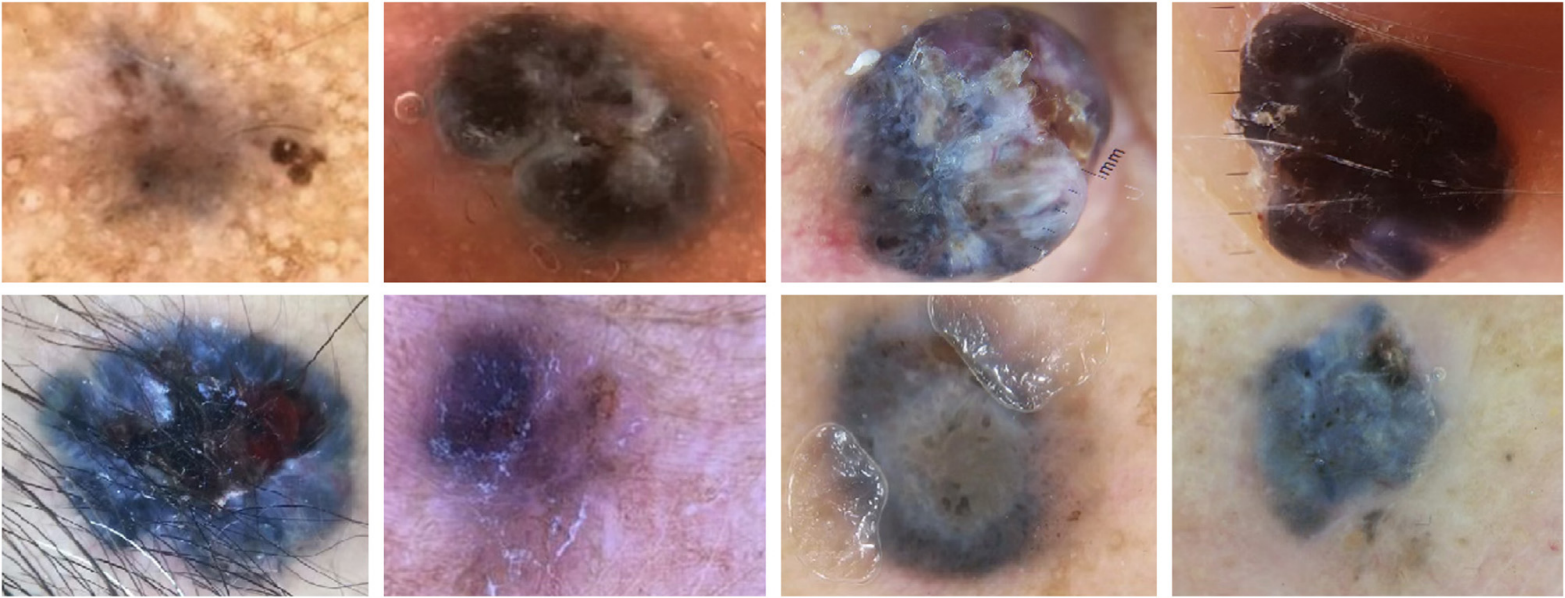
Diffusely pigmented basal cell carcinomas ‒ lesions with extensive pigmentation masking classical dermoscopic features.

Differences between the more prevalent dermoscopic structures in nodular and superficial BCCs were identified, corroborating previous studies.^10^ Nodular BCCs had large blue-grey ovoid nests and arborizing telangiectasias, whereas superficial BCCs more often presented multiple small erosions, leaf-like structures, spoke-wheel structures, and a white/red structureless background. These findings are consistent with those described by Reiter et al.,^10^ confirming the ability of dermoscopy to predict BCC subtype even in darker skin.

Study limitations include its retrospective design, potential selection bias due to inclusion of clinically suspicious lesions, and subjectivity in both phototype classification and dermoscopic interpretation. To minimize these biases, three dermatologists jointly evaluated all images and reached a consensus.

In conclusion, most BCCs in phototypes IV and V are pigmented, often diffusely, which may mask classical diagnostic features. Leaf-like areas represent the most frequent dermoscopic structure, whereas vascular patterns are less visible. Recognizing these distinctive features is essential to improve diagnostic accuracy in skin of color. Expanding research to include more diverse populations will help refine dermoscopic criteria and promote greater equity in dermatologic care.

## Authors' contributions

Priscila Jordana Costa Valadares: Critical literature review; data collection, analysis and interpretation; preparation and writing of the manuscript; study conception and planning.

Maria Luisa Pires de Freitas: Data collection, analysis and interpretation.

Diego Guimarães Florêncio Pujoni: Statistical analysis.

Flávia Vasques Bittencourt: Data collection, analysis and interpretation; effective participation in research orientation; manuscript critical review; study conception and planning.

## Ethics statement

Universidade Federal de Minas Gerais IRB approval #5137559.

## Financial support

None declared.

## Research data availability

The entire dataset supporting the results of this study was published in this article.

## Conflicts of interest

None declared.

## References

[1] Cameron M.C., Lee E., Hibler B.P., Barker C.A., Mori S., Cordova M. (2019). Basal cell carcinoma: epidemiology, pathophysiology, clinical and histological subtypes, and disease associations. J Am Acad Dermatol.

[2] Karampinis E., Lallas A., Lazaridou E., Errichetti E., Apalla Z. (2024). Clinical and dermoscopic patterns of basal cell carcinoma and its mimickers in skin of color: a practical summary. Medicina.

[3] Higgins S., Nazemi A., Chow M., Wysong A. (2018). Review of nonmelanoma skin cancer in African Americans, Hispanics, and Asians. Dermatol Surg.

[4] Salmen N.L., Kunz M., Osella-Abate S., Marghoob A.A., Lallas A. (2024). Skin color reporting in basal cell carcinoma-related randomized controlled trials in top dermatology journals: a systematic review. Arch Dermatol Res.

[5] Wolner Z.J., Bajaj S., Flores E., Carrera C., Navarrete-Dechent C., Dusza S.W. (2018). Variation in dermoscopic features of basal cell carcinoma as a function of anatomical location and pigmentation status. Br J Dermatol.

[6] Manci R., Dauscher M., Marchetti M.A., Usatine R.P., Rotemberg V., Dusza S. (2022). Features of skin cancer in black individuals: a single-institution retrospective cohort study. Dermatol Pract Concept.

[7] Menzies S.W., Westerhoff K., Rabinovitz H., Kopf A.W., McCarthy W.H., Katz B. (2000). Surface microscopy of pigmented basal cell carcinoma. Arch Dermatol.

[8] Behera B., Kumari R., Thappa D.M., Gochhait D., Srinivas B.H., Ayyanar P. (2023). Dermoscopic features of basal cell carcinoma in skin of color: a retrospective cross-sectional study from Puducherry, South India. Indian J Dermatol Venereol Leprol.

[9] Soughi M., Meziane M., Gallouj S., Mernissi F. (2019). Descriptive dermoscopic study of basal cell carcinoma in Morocco. Pan Afr Med J.

[10] Reiter O., Mimouni I., Dusza S.W., Halpern A.C., Leshem Y.A., Marghoob A.A. (2021). Dermoscopic features of basal cell carcinoma and its subtypes: a systematic review. J Am Acad Dermatol.

